# Severe Acute Respiratory Syndrome, Beijing, 2003

**DOI:** 10.3201/eid1001.030553

**Published:** 2004-01

**Authors:** Wannian Liang, Zonghan Zhu, Jiyong Guo, Zejun Liu, Xiong He, Weigong Zhou, Daniel P. Chin, Anne Schuchat, for the Beijing Joint SARS Expert Group

**Affiliations:** * Beijing Municipal Health Bureau and Beijing Municipal Centers for Disease Prevention and Control, Beijing, China; † Centers for Disease Control and Prevention, Atlanta, Georgia, USA; ‡ World Health Organization, Beijing, China

**Keywords:** severe acute respiratory syndrome, disease outbreaks, epidemiology, China, nosocomial infection, SARS virus, disease transmission

## Abstract

The largest outbreak of severe acute respiratory syndrome (SARS) struck Beijing in spring 2003. Multiple importations of SARS to Beijing initiated transmission in several healthcare facilities. Beijing’s outbreak began March 5; by late April, daily hospital admissions for SARS exceeded 100 for several days; 2,521 cases of probable SARS occurred. Attack rates were highest in those 20–39 years of age; 1% of cases occurred in children <10 years. The case-fatality rate was highest among patients >65 years (27.7% vs. 4.8% for those 20–64 years, p < 0.001). Healthcare workers accounted for 16% of probable cases. The proportion of case-patients without known contact to a SARS patient increased significantly in May. Implementation of early detection, isolation, contact tracing, quarantine, triage of case-patients to designated SARS hospitals, and community mobilization ended the outbreak.

By July 4, 2003, a total of 8,439 probable cases and 812 deaths from severe acute respiratory syndrome (SARS) had been identified from 30 countries (URL: http://www.who.int/csr/sars/en/). A novel coronavirus (SARS-CoV) was found to be the cause of this multicountry outbreak (*1*–*3*). Most cases of SARS occurred in China, where the virus apparently emerged first, most likely from animal sources. The largest outbreak of SARS occurred in Beijing.

In Beijing, the SARS outbreak was reported in April 2003, against a backdrop of earlier outbreaks detected in Guangdong, Hong Kong (*4*,*5*), Hanoi, Toronto (*6*), and Singapore (*7*). In contrast to Toronto, where the entire outbreak originated from a single importation (*6*), Beijing’s outbreak involved multiple distinct imported cases, and transmission from index cases was amplified within several healthcare facilities. Widespread transmission came under control after Beijing municipal authorities aggressively implemented measures to enhance detection, isolate case-patients, and trace contacts to minimize further opportunities for transmission in community and institutional settings. This report summarizes the descriptive epidemiology of Beijing’s outbreak and the emergency interventions that were implemented to control the local situation.

## Methods

### Setting

Beijing municipality has an estimated population of 13.8 million and includes 14 districts and four counties. Approximately 85,000 healthcare workers live there. Disease reporting and epidemic investigations of reported cases were conducted through the collaboration of the Beijing Center for Prevention and Disease Control and district centers within Beijing, using guidelines for surveillance and case investigation issued by China’s Ministry of Health.

### Case Definitions

China established a case definition for “infectious atypical pneumonia,” also termed SARS, with minor modifications implemented during the course of the outbreak. After May 3, probable (“clinically confirmed”) and suspected cases were defined according to 1) epidemiologic history (either contact with other SARS patients or exposure to a SARS-affected area); 2) symptoms and signs of fever and respiratory illness; 3) normal or decreased leukoctye count; 4) chest radiograph abnormalities; and 5) absence of substantial improvement with antibiotic treatment (Table 1). We have included cases reported as probable according to the case definition in place at the time of report, consistent with a strategy used by other investigators (*8*).

**Table 1:** Case definition for severe acute respiratory syndrome (infectious atypical pneumonia) in China as of May 3, 2003

Category	Criteria^a^
Probable	1.1 + 2 + 4, or 1.2 +2+4+5, or 1.2+2+3+4
Suspected	1.1+2+3 or 1.2+2+4 or 2+3+4
Under medical observation	1.2+2+3

^a^1. Epidemiologic history: 1.1:Having close contact with a patient, or being a member of infected cluster, or having infected other persons; 1.2: Having visited or resided in cities or areas where SARS cases were reported with secondary transmission during the 2 weeks before onset of disease. 2. Symptoms and signs of febrile respiratory illness. 3. Normal or decreased leukocyte count. 4. Chest x-ray changes. 5. Lack of response to antibiotic treatment.

### Case Reporting

 When a possible case-patient is identified in a healthcare facility within Beijing, a panel of experts at the facility reviews clinical information to classify the illness as probable, suspected, or under observation. Case classification is updated on the basis of clinical progression and availability of alternative diagnoses to account for the illness, although diagnostic testing for other agents was not extensive during most of the epidemic. During May, clinical experts were dispatched to SARS hospitals to improve how consistently cases were classified according to the national case definition. For probable and suspected cases, healthcare providers complete a standard report form, which is faxed to the relevant district center for disease control. A district epidemic investigator then interviews the patient (or family member) and completes a standardized epidemic investigation form regarding demographic and clinical data, as well as the patient’s contacts within the 2 weeks before symptom onset, in an attempt to identify the patient’s source of infection. The district is responsible for identifying persons who had contact with the patient between the onset of symptoms and hospitalization. Those who had close contact are placed under home medical observation by community health center personnel and are quarantined to restrict their circulation in the broader community.

### Laboratory Testing

 Serum was collected from patients at certain hospitals for detection of anti–SARS-CoV antibodies by using one of two locally developed enzyme-linked immunosorbent assay (ELISA) kits; one was developed by the Beijing Genomics Institute in partnership with the Academy of Military Medical Sciences, and the other was developed by the China Center for Disease Control. SARS-CoV was also isolated from selected clinical specimens; substantial partial genome sequencing for four Beijing strains (AY278488, AYAY278487, AY278490, and AY279354) was submitted to GenBank April 17–April 19, 2003, by E. Qin et al. from the Academy of Military Medical Sciences and the Beijing Genomics Institute in Beijing. Details on laboratory tests are reported separately (*9*,*10*).

### Data Analysis

 Data were entered into either a Microsoft Excel database (case report forms) or an Oracle database (detailed epidemiologic investigation forms). Data analysis used SPSS (SPSS Inc., Chicago, IL) software. Chi-square or, when appropriate, Fisher exact test was used for comparison of proportions. Because date of onset was missing for 985 (26.8%) of the 3,665 patients with probable and suspected cases reported through May 20, we present temporal information based on date of hospitalization, which was missing in 155 (4.2%) of case-patients.

## Results

### Importation Phase

The earliest cases in Beijing occurred in persons who were infected with SARS in Guangdong and Hong Kong.

#### Index Case 1

**Figure 1:**
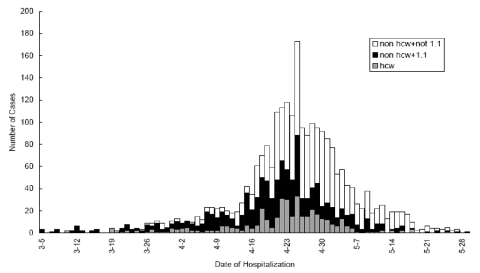
Epidemic curve—severe acute respiratory syndrome (SARS) probable case-patients by date of hospitalization and type of exposure, Beijing, 2003. Open bars indicate nonhealthcare workers without contact with a SARS patient; dark bars (“1.1”) indicate nonhealthcare workers with contact with a SARS patient; light filled bars indicate healthcare workers.

The first apparent case of SARS in Beijing was identified on March 5 in a 27-year-old businesswoman in whom symptoms developed on February 22 while she was traveling in Guangdong (Figure 1). She sought medical attention in Shanxi Province, where SARS subsequently developed in two doctors and a nurse who cared for her. After she returned to Beijing, she was hospitalized in a military hospital, then transferred to an infectious disease hospital. SARS developed in 10 healthcare workers exposed at the two Beijing hospitals as well as 8 of the patient’s family members and close colleagues or friends. Both of the patient’s parents died from SARS. Healthcare workers cared for the patient before SARS was suspected and used no personal protective equipment.

#### Index Case 2

A 72-year-old man visited a relative in Hong Kong’s Prince of Wales Hospital, and symptoms developed on March 14, 2003. On March 15, the patient flew from Hong Kong to Beijing on China Air flight 112. He was evaluated in one hospital on arrival in Beijing but was not admitted. The next day, his family brought him to a second Beijing hospital, where after a successful resuscitation in the emergency department, he was admitted to the hospital. He died there on March 20. Contact tracing and epidemic investigation suggest that at least 59 SARS cases in Beijing can be traced back to this patient, including illness in three members of his immediate family, in six of seven healthcare workers who assisted in the emergency room resuscitation, and in one other healthcare worker in the facility. The remaining cases occurred in other patients and their contacts. In addition to Beijing cases, transmission on this airplane flight has been linked to SARS cases in other areas, including Taiwan and Inner Mongolia. Besides these two index case-patients, several later SARS case-patients in Beijing had traveled to other affected areas before the onset of clinical symptoms.

### Amplification in Healthcare Facilities

**Figure 2:**
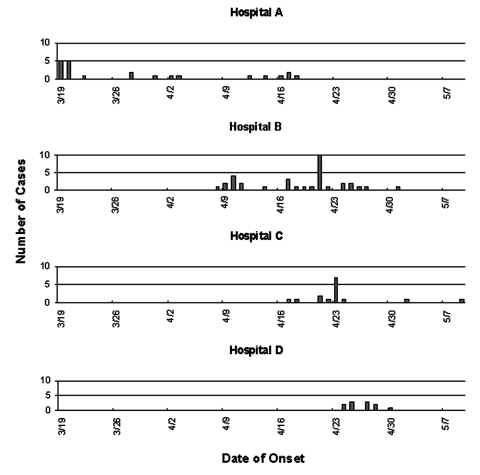
Clusters of severe acute respiratory syndrome (SARS) cases among healthcare workers in four hospitals, Beijing 2003.

 SARS occurred in healthcare workers in >70 hospitals throughout Beijing, and clusters of >20 probable SARS cases among healthcare workers occurred in four Beijing hospitals (Figure 2). Apparent transmission of SARS within fever clinics and selected hospitals prompted closure of four hospitals and numerous fever clinics. One large hospital, where 41 probable cases occurred among healthcare workers and numerous cases occurred among patients and contacts, was closed on April 23. SARS patients were transferred to designated SARS hospitals, and the remaining patients, staff, and visitors were quarantined in the hospital for 2 weeks.

### Evolution of Outbreak

Through June 2003, a total of 2,521 patients with probable cases of SARS were hospitalized in Beijing. The outbreak peaked during the 3rd and 4th weeks in April, when hospitalizations for probable SARS exceeded 100 cases for several days, and an increased proportion of case-patients reported having no known contact with a SARS patient (Figure 1).

### Description of Cases

Of 2,521 probable SARS cases in Beijing, 2,444 (96.7%) cases reported by May 20 had data available for review and constitute the remainder of this report. Of the 2,444 probable case-patients, 1,009 (41.3%) had a history of close contact with a patient with SARS; 395 (16.2%) of the probable cases occurred in healthcare workers. Overall, 42.9% of probable case-patients had no previous contact with a SARS case-patient or travel to affected areas outside Beijing. However, the proportion of probable case-patients with no direct contact with a SARS patient increased from 50.7% for case-patients who were hospitalized before May 1, to 75.2% for those admitted to hospitals in May (p < 0.001). Among probable SARS case-patients who were hospitalized in March and April, healthcare workers accounted for 18.7% (n = 329), compared with 10.7% (n = 61) for case-patients who were hospitalized on May 1 or thereafter (p < 0.001)*.*

**Figure 3:**
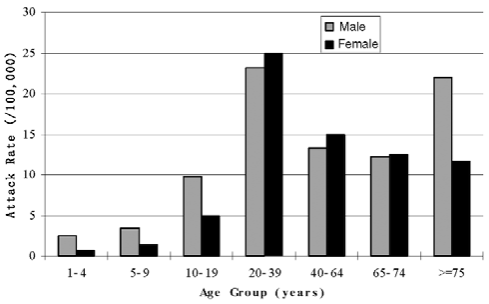
Attack rates (cases per 100,000 population) by age and sex of probable severe acute respiratory syndrome (SARS), Beijing 2003.

The demographic characteristics of case-patients with probable SARS are shown in Table 2. Children <10 years of age accounted for 0.9% of probable cases, and the median age of those who became ill was 33 years. Age-specific attack rates were highest in those 20–39 years of age (relative risk [RR] 1.7, 95% confidence interval (CI) 1.53 to 1.89, compared with those 40–64years, and significantly lower in children (1–4 years of age, RR 0.12 [CI 0.05 to 0.28], 5–9 years, RR 0.17 [CI 0.09 to 0.31] and 10–19 years, RR 0.53 [CI 0.44 to 0.64], compared with those aged 40–64 years). Overall, male patients had similar rates as female patients, but the risk differed significantly in certain age groups: among those 10–19 years of age, the RR for SARS in male patients was 1.96, 95% CI 1.36 to 1.83, compared with that of females; and in those >75 years, RR for male patients was 1.88 (95% CI 1.08 to 3.29) (Figure 3). The attack rate for probable SARS among healthcare workers in Beijing is estimated as 465 per 100,000. Consistent with the case definitions in use in Beijing during the outbreak, chest x-ray changes were evident in >85% of probable case-patients. As of May 20, the case-fatality rate was 6.4% for probable SARS case-patients. Case-fatality rates increased with age (0.5% in <20 year olds; 4.8% for those 20–64 years; and 27.7% for >65 years of age, p < 0.001). By June 16, 2003, a total of 190 deaths among 2,521 probable SARS case-patients were reported from Beijing, and 2,053 patients had been discharged from the hospital. The case fatality rate among probable case-patients, excluding those still hospitalized, was 8.4%.

**Table 2:** Characteristics of probable cases of severe acute respiratory syndrome (SARS) in Beijing, 2003

Characteristic^a^	Probable case-patients; N (%)
**Demographic** Male sex	1,217/2,406 (50.6)
Age (y)	
1–4	6/2,397 (0.2)
5–9	17/2,397 (0.7)
10–19	165/2,397 (6.9)
20–39	1,270/2,397 (53.0)
40–64	733/2,397 (30.6)
65–74	147/2,397 (6.1)
>75	59/2,397 (2.5)
Median age (range)	33 (1–93)
Fatal outcome	156/2,444 (6.4)
Healthcare worker	395/2,444(16.2)
**Admission symptoms**	
Fever	1,646/1,693 (97.2)
Cough	749/1,693 (44.2)
Difficulty breathing	166/1,693 (9.8)
Chest tightness	331/1,693 (19.6)
Diarrhea	189/1,693 (11.2)

^a^Information was not available for the sex of 38 probable case-patients and for the age of 47 probable case-patients reported through May 20.

According to clinical information available on the case investigation form, nearly all SARS case-patients had the initial symptom of fever, and many had a cough (44.2%), but only 11.0% had diarrhea. Mean leukocyte count on admission was 5.5 x 10^9^/L, and 25.2% had leukocyte counts <4.0 x 10^9^/L (the normal limit).

 For the 1,009 probable SARS case-patients with reported contact with another SARS patient, the most recent date of such contact was collected; 595 of these patients had onset dates available, permitting approximation of the shortest possible incubation period. Among these patients, a mean of 7.8 days (median 6) occurred from most recent exposure to onset of symptoms.

### Control Measures

Prompted by the rapid expansion of the epidemic from April 16 to April 19, the Beijing Municipal Government established a Joint SARS Leading Group to oversee crisis management through 10 task forces. The medical and public health task force set up an emergency command center on April 24 and organized fever clinics for triage, designated SARS areas within hospitals for isolation and specialized care, provided personal protective equipment and training for healthcare workers, and introduced community-based prevention and control through case detection, isolation, quarantine, and community mobilization. To reduce transmission within healthcare settings, Beijing authorities issued protocols for triage, isolation, case management, and administrative controls, which prohibited visitors to hospitals and separated patients who were under medical observation or suspected of having SARS from areas with other patients.

The medical emergency command center included teams for clinical diagnosis and treatment, critical care, patient transport, infection control, and information management. Local shortages of isolation rooms, intensive care facilities, and hospital beds were addressed by dispatching specially equipped ambulances to transfer SARS patients to designated facilities. An anticipated shortage of hospital beds for care and isolation of SARS patients prompted authorities to construct a new 1,000-bed hospital in 8 days.

 On April 27, all patients with probable cases of SARS were moved to designated areas within hospitals. At one point, 27 municipal and 21 district hospitals were providing care to SARS patients. On May 8, 2003, the medical and public health task force finished concentrating all the probable case-patients into 16 designated municipal hospitals, with 30 district hospitals providing care for patients with suspected SARS. More than 60 fever clinics were established throughout the city to triage patients with acute febrile illness, permitting prompt isolation of patients who required further observation and referral to the appropriate level of care to rule out SARS. By June 19, 2003, a total of 30,172 people who had had close contact with probable or suspected SARS case-patients had been quarantined separately or in groups for 2 weeks after their last exposure to a SARS case-patient.

In addition to interventions directed at managing patients, their contacts, and healthcare facilities, schools were closed, travel was restricted, the community was educated about seeking care at designated sites, and temperatures were monitored at frequent check points. Service professionals were required to wear masks, and many community members donned masks as well.

### Laboratory Confirmation

SARS-CoV was isolated from many patients in Beijing, and sequences from four Beijing isolates (source: GenBank) were compared with strains from other areas (*11*). The cause of infection was confirmed for a series of patients with severe illness who were cared for at Ditan Hospital, by using an ELISA developed by the China Center for Disease Control. Among 164 case-patients with probable SARS (who had severe illness) tested by mid-May, 98% had SARS-CoV–specific immunoglobulin (Ig) G detected from samples collected >35 days after illness onset; 55% had SARS-CoV–specific IgG detected 16–21 days after symptom onset, and 82% by 22 to 28 days after illness onset.

## Discussion

Beijing experienced the largest outbreak of SARS yet recognized. The disease was transported to Beijing by multiple travelers. One case-patient originated in Guangdong and transported the virus to Shanxi along the way, and a second case-patient appears to have acquired the virus during a hospital visit in Hong Kong. He then transmitted the virus to other travelers encountered while he had symptoms on the return airline flight to Beijing and to those he came into contact with in a hospital in Beijing. The imported cases initiated cascades of illness among the family members, healthcare workers, and other hospitalized patients. Patients seeking care in multiple facilities and clinicians failing to recognize nonspecific respiratory symptoms as indications for isolation and use personal protective equipment permitted the efficient transmission of the virus to numerous healthcare workers, patients, and others throughout Beijing.

Amplification of transmission in healthcare settings was likely enhanced by resuscitation of one of the index patients in an emergency department. Since SARS patients were initially cared for on general medical wards, the virus was transmitted to other patients hospitalized for unrelated conditions; such persons may be more vulnerable to infection as well as severe outcomes (*8*,*12*,*13*). Designation of SARS wards and later specialized SARS hospitals facilitated control of transmission within healthcare settings in Beijing. However, the delay before these steps were taken permitted numbers of infected persons to increase in healthcare settings, which likely permitted transmission to community members through visits to hospitals, before such visits were stopped and strict isolation measures observed.

Classifying patients as having probable or suspected SARS may be difficult with the current case definitions. Although a novel coronavirus was rapidly discovered as the cause of SARS, this disease appeared to both clinicians and public health workers as a nonspecific clinical syndrome. The clinical features have substantial overlap with those caused by common respiratory viruses and bacteria. Implementation of the case definition on the basis of nonspecific clinical features was particularly challenging in areas with community transmission, since the ability to focus surveillance and isolation efforts on persons with epidemiologic links to specific travel or contact with other SARS patients was no longer appropriate. Although the case definition used by China included some components to enhance specificity (i.e., normal or low leukocyte count, lack of response to antibiotics), the definition remains nonspecific. Future surveillance and case management will benefit greatly from incorporating laboratory tests, particularly if sufficiently sensitive laboratory methods can be developed which are amenable to point-of-care use early in the clinical course. In the meantime, available assays for SARS-CoV, including ELISA and polymerase chain reaction testing, will be valuable complements to epidemiologic surveillance for understanding recent disease patterns. Elimination of SARS as a public health threat will require major commitment to laboratory testing of possible case-patients.

The age-specific attack rates of SARS in Beijing support findings from other outbreaks of SARS. High rates among those 20–39 years of age likely reflect disease among healthcare workers with occupational exposures, and high rates in the elderly may represent patients with nosocomial acquisition. The extremely low rate observed in children in Beijing is noteworthy. Pediatric cases of SARS have also been relatively rare in other countries (*14*), but since most other outbreaks remained concentrated at healthcare facilities, this pattern could simply reflect limited exposure to ill patients, thought to be the most efficient transmitters. Given the size of Beijing’s outbreak and the spread beyond hospitals, absence of exposure is unlikely to fully account for the low incidence of SARS in children. Whether asymptomatic or mild infection is more likely to develop in children, and whether children are able to transmit the virus to others in the absence of clinical illness, are important questions which must be addressed to guide control.

The steep increase in cases of SARS in late April posed a major challenge to Beijing’s healthcare and public health systems. The magnitude of ill healthcare workers, incident cases, and affected facilities necessitated a strategic response. Shortages of beds and isolation rooms, as well as ongoing transmission in some hospitals, prompted the designation of selected hospitals for SARS patients; the preparation of special protocols for care, isolation, and healthcare worker protection; and ultimately, the construction of a large new facility to ensure that capacity could keep up with projected demand. More than 30,000 persons were placed under quarantine. Maintaining adequate case investigation challenged local public health staff, and the relatively high proportion of cases with no reported contact with a SARS patient may reflect limitations in the methods of identifying exposures during the epidemic’s peak, rather than the absence of an identifiable source in all of these cases. We are reevaluating potential sources of SARS among patients initially reported to have no contact with a SARS patient (*15*). Given the nonspecific case definition, some of these patients may not have been infected with SARS-CoV, and we are also further evaluating this possibility. By restricting community gatherings and travel, closing schools and entertainment venues, major reductions in social contact also may have contributed to slowing transmission. The time from symptom onset to hospitalization became shorter during the course of the response efforts (before April 21, median 6 days vs. median 2 days thereafter); faster recognition of the condition and isolation of patients were likely factors in bringing the epidemic under control. Because multiple interventions were instituted simultaneously, distinguishing the effectiveness of isolation and quarantine measures from the impact of broader measures implemented for the general community will be difficult. Nevertheless, evaluation of several of these efforts is in progress.

A principal lesson learned from the Beijing experience is the importance of rapid response to SARS. Early detection of patients and prompt isolation can limit transmission, and adherence to personal and administrative infection control measures can reduce opportunities for transmission within healthcare facilities. The resources needed to respond to simultaneous outbreaks in multiple hospitals and address community transmission are much greater than those required for individual case investigation or management. Communities seeking to prepare for SARS must be alert to the speed with which one imported case can lead to dozens or hundreds of transmission chains. Thus a strong system of early detection and open communication will facilitate prompt recognition of possible problems and immediate response measures. Addressing community concerns, including fear of attending fever clinics or stigma associated with having one’s neighborhood quarantined, should be an important component of planning efforts.

 The Beijing epidemic has many features in common with those experienced elsewhere, including the disproportionate impact on healthcare workers and amplification of disease in hospitals. The pattern of transmission is consistent with droplet or contact spread. The apparent success of infection control, isolation, contact tracing, and quarantine in bringing the outbreak under control is encouraging, particularly because these efforts were introduced later in the epidemic in Beijing than in some other settings. Whether features particular to Beijing had a major influence on the evolution or characteristics of the outbreak is not yet clear. Careful clinical assessments of patients cared for during the outbreak will be valuable, since in addition to steroids and antiviral drugs, traditional Chinese medicine was frequently used in caring for SARS patients. Whether treatment strategies might be responsible for the lower age-specific case-fatality ratios in Beijing compared with reports from other places is not yet known, and the lower case-fatality ratio may derive in part from the nonspecific case definition with resulting misclassification of some pneumonias of other causes as probable SARS. While population density might have made the outbreak more difficult to control in Beijing, the massive and efficient mobilization of communities and health workers to respond to the outbreak was likely an asset.

In the response to SARS, opportunities exist for ensuring broad public health benefits. A stronger public health infrastructure capable of improved preparedness and response to SARS will also improve control of other diseases. Strengthening infection control practices to prevent repeated introductions of SARS epidemics is likely to reduce other healthcare-associated infections. Like other emerging infectious diseases, SARS has demonstrated the importance of enhanced communication between disparate geographic regions and diverse sectors of society.
